# Dichotomy in Neutralizing Antibody Induction to Peptide-Conjugated Vaccine in Squalene Emulsion Contrast With Aluminum Hydroxide Formulation

**DOI:** 10.3389/fimmu.2022.848571

**Published:** 2022-04-07

**Authors:** Olivia Bonduelle, Chloé Chaudesaigues, Monica Tolazzi, Ehsan Suleiman, Simon de Bernard, Karine Alves, Julien Nourikyan, Mylene Bohec, Laura G. Baudrin, Dietmar Katinger, Patrice Debré, Gabriella Scarlatti, Vincent Vieillard, Behazine Combadière

**Affiliations:** ^1^ Sorbonne Université, Institut national de la santé et de la recherche médicale (Inserm) U1135, Centre d’Immunologie et des Maladies Infectieuses, Paris, France; ^2^ Viral Evolution and Transmission Unit, Division of Immunology, Transplantation and Infectious Diseases, Istituto di Ricovero e Cura a Carattere Scientifico (IRCCS) San Raffaele Scientific Institute, Milan, Italy; ^3^ Polymun Scientific Immunbiologische Forschung GmbH, Klosterneuburg, Austria; ^4^ Altrabio, Lyon, France; ^5^ Institut Curie, Genomics of Excellence (ICGex) Platform, Paris Science et Lettres (PSL) Research University, Paris, France

**Keywords:** adjuvant, B-cells, neutralizing, vaccine, squalene

## Abstract

W614A-3S peptide is a modified 3S motif of the HIV-gp41 (mutation W614A). We previously detected the presence of natural neutralizing antibodies directed against W614A-3S peptide (NAbs) in long-term non-progressor HIV^+^ patients. Here, we compared the efficacy of W614A-3S peptide formulated in either squalene emulsion (SQE) or in aluminum hydroxide (Alum) in inducing broadly-NAbs (bNAbs). Rabbit and mouse models were used to screen the induction of bNAbs following 4 immunizations. SQE was more efficient than Alum formulation in inducing W614A-3S-specific bNAbs with up to 67%–93% of HIV strains neutralized. We then analyzed the quality of peptide-specific murine B cells by single-cell gene expression by quantitative reverse transcription-PCR and single-cell V(D)J sequencing. We found more frequent germinal center B cells in SQE than in Alum, albeit with a different gene expression profile. The V(D)J sequencing of W614A-3S-specific BCR showed significant differences in BCR sequences and validates the dichotomy between adjuvant formulations. All sixteen BCR sequences which were cloned were specific of peptide. Adjuvant formulations of W614A-3S-peptide-conjugated immunogen impact the quantity and quality of B cell immune responses at both the gene expression level and BCR sequence.

## Introduction

The disease caused by HIV remains a major public health problem. Although there is no cure for HIV infection, effective antiretroviral therapy aids in controlling the virus and helps to prevent transmission. HIV poses challenges for vaccine development. Over time, the virus has evolved a number of evasion mechanisms, particularly through the Env protein, by extensive amino acid variation and conformational instability. HIV-1 Env is the only target of antiviral neutralizing antibodies (NAbs) (1). Ten percent to 30% of HIV^+^ patients have serum antibodies capable of neutralizing virus infection of cells, with some also able to neutralize a majority of different cross-clade viral strains (2). The first challenge is to design a vaccine formulation that would induce Abs-neutralizing virus entry against large multi-clade panels of genetically diverse HIV-1, to maximize potential clinical benefit. However, Env evolution creates a major hurdle for vaccine design (3, 4). The HIV-1 Env is a heavily glycosylated trimeric protein comprising three identical surface gp120 molecules, each non-covalently associated with a transmembrane gp41 molecule (3). The gp41 molecule is involved in the final steps of viral envelope fusion to the host cell membrane (5) and has been proposed in vaccination strategies (6, 7).

We previously described a specific and highly conserved motif of HIV-1 gp41, named 3S “NH2-pwnaSWSNKSlddiw-COOH” (namely, 3S motif), which is absent in HIV-2 and SIV (8). A mutated form of the 3S motif, with a single amino acid change at position W614 to alanine (NH2-pwnaSASNKSlddiw-COOH; namely W614A-3S), had increased immunogenicity in preclinical models. Animal models including mice, rabbits, and macaques showed immunogenicity of the W614A-3S peptide when coupled with carrier protein and adjuvanted in incomplete Freund adjuvant (IFA), a water-in-oil emulsion (9). Importantly, natural Abs against W614A-3S eluted from the plasma of HIV-1 patients showed neutralizing activity and were detected exclusively in patients with high CD4 counts and undetectable (<20 copies/ml) or controlled viral loads (9, 10). W614A-3S Abs were detected in 23.5% (16/68) of untreated long-term non-progressor (LTNP) patients compared with <5% of HIV-1 progressor patients. The neutralizing capacity of W614A-3S Abs was inversely correlated with viral load and was associated with the preservation of high CD4+ T-cell counts and T-cell responses. In LTNP patients, the level of W614A-3S NAbs was significantly correlated with CD4+ T-cell counts at 1 year and even more at 5 years after inclusion (11).

Translation into human clinical trials requires validation of vaccine formulations in preclinical studies. First, W614A-3S were conjugated to a carrier protein, Cross-Reacting Material 197 (CRM197). CRM197 is a genetically detoxified form of diphtheria toxin. A single mutation at position 52, substituting glutamic acid for glycine, causes the ADP-ribosyltransferase activity of the native toxin to be lost (12). CRM197 is used as a carrier protein in a number of approved conjugate vaccines (13, 14). Second, we proposed to use clinical authorized adjuvant formulation such as squalene emulsion (SQE) (MF59-Like) formulation or aluminum hydroxide (Alum) to test the ability of W614A-3S formulations to induce broadly NAbs. We sought to elucidate the mechanism of B cell maturation by single-cell gene expression. We demonstrated the dichotomy between SQE-based adjuvant and Alum in the induction of lymph node germinal center (GC), leading to NAbs versus non-NAbs, respectively. Single B-cell analyses validated this dichotomy, showing differential gene expression as well as BCR sequences of W614A-3S-specific B cells generated in SQE compared to Alum formulations.

## Materials and Methods

### Animals

BALB/cByJ female mice were purchased from Janvier Labs (Le Genest-Saint-Isle, France), housed at a free animal facility (Centre d’Experimentation Fonctionnelle (CEF) of Sorbonne University, France) under specified pathogen-free conditions, and used for experiments at 6–10 weeks of age. New Zealand rabbits were housed at Covalab company (Bron, France).

### Immunizations and Vaccine Antigens

Rabbits received 50 μg of the peptide W614A-3S coupled with CRM197 by the intramuscular route at Week 0 (W0), W2, W4, and W10. Mice received 10 μg of peptide coupled with keyhole limpet hemocyanin (KLH; Covalab) carrier protein or 11.7 μg of W614A-3S peptide coupled with CRM197 (Minka Therapeutics, Ile-de-France, France) by intramuscular injections (in both quadriceps of left and right thighs) at the same kinetic (or W18 for mouse CRM197 experiment). W614A-3S peptide (NH2-PWNASASNKSLDDIW-COOH) is a mutated peptide of HIV-1 gp41 (9). The antigen was administered with PBS1X (Life Technologies, Carlsbad, CA, USA), aluminum hydroxide (Alum; 100 μg for mouse and 250 μg for rabbit; Alhydrogel 2.0%; InvivoGen, San Diego, CA, USA), or squalene emulsion (SQE; 2.5% v/v for mouse and rabbit; Polymun, Klosterneuburg, Austria).

### Antibody Detection

Sera were collected before first injection and at different time points after each immunization (W4, W6, W10, W12, W14, and W20 for rabbits; W3, W5, W8, and W20 for mice) and stored at -80°C prior to evaluation of W614A-3S-specific IgG titration by ELISA. MaxiSorp plates (Corning, Tewksbury, MA, USA) were coated overnight with 50 ng/well of W614A-3S peptide (Covalab). For W614A-3S-monoclonal Ab evaluation, plates were coated with 50 ng/well of W614A-3S-KLH vaccine. Wells were saturated with PBS1X + 1% bovine serum albumin (Life Technologies) prior to serum serial dilutions of 1/10 or different dilutions of W614A-3S-monoclonal Abs (32,000 to 0.97 ng/ml). All samples were tested in duplicate. Peptide-specific Abs were revealed by horseradish peroxidase (HRP)-conjugated anti-mouse IgG (1/10,000; SouthernBiotech, Birmingham, AL, USA) or detection antibody biotinylated anti-rabbit IgG (1/5,000; Jackson ImmunoResearch, West Grove, PA, USA) followed by HRP-streptavidin (1/200; R&D Systems, Minneapolis, MN, USA). Enzymatic activity was measured by adding tetramethylbenzidine (TMB; Pierce Endogen) and stopped by 1 N H_2_SO_4_. Optical density (OD) of each well was monitored at 450 nm with a FlexStation 3 ELISA reader (Molecular Devices, Eugene, OR, USA) and Ascent Software version 2.6. EC50 was calculated by sigmoidal dose response (Prism 9 software). For the NAb assay, serum-purified W614A-3S-specific Abs were obtained as described (9). A pool of 4–5 sera was used for mouse purified NAb evaluation. Viral titration of infectious molecular clones or pseudotyped viruses and TZMbl neutralization assays were performed as previously described (15). Briefly, duplicates of six steps of 3-fold dilution, starting with 1:20 of each serum or 5 μg/ml of peptide-specific purified Abs, were incubated with viral supernatant (at relative luminescence units (RLU) between 150,000 and 200,000) for 1 h. Thereafter, 10^4^ TZM-bl cells were added, and plates were incubated for 48 h at 37°C, when the Bright-Glo Luciferase assay system (Promega, Madison, WI, USA) was added to measure luciferase activity with a Mithras luminometer (Berthold, Bad Wildbad, Germany). Positive controls were monoclonal Abs with known neutralizing titers. Neutralization titers were defined as the sample dilution at which RLU were reduced by 50% compared to virus control wells after subtraction of background RLU in control wells with only cells. Inhibitory concentrations (IC) 50 were calculated with a linear interpolation method using the mean of the duplicate responses (15). Neutralizing IC50 of control monoclonal Abs, binding to different Env regions, is shown in **Supplemental Figure 3**.

### Cell Phenotype and Isolation

Draining lymph nodes (dLN; inguinal) were harvested 7 days after each immunization. Cells were stained with W614A-3S coupled with biotinylated ovalbumin (Ova) protein (Covalab) for 30 min at room temperature before cell surface antigen staining with a standard method after receptor Fc blocking with CD16/CD32 (clone 2.4G2; BD Biosciences, San Jose, CA, USA), and the following anti-mouse Abs: CD3e (clone 145-2C11; eBioscience, San Diego, CA, USA), CD45R/B220 (clone RA3-6B2), CD19 (clone 1D3), IgG1 (clone A85-1), IgD (clone 11-26c.2a), T- and B-cell activation antigen (clone GL7), and streptavidin (BD Biosciences). For cell analysis, dead cells were excluded by using the LIVE/DEAD fixable kit (Molecular Probes, Eugene, OR, USA). Cells were analyzed by BD LSRFortessa flow cytometry or isolated by BD FACSAria II sorter. A pool of five mice per condition at W3 and W5 was used for W614A-3S-specific B cell isolations (BioMark Dynamic array). A pool of 25 mice per condition at W11 was used for W614A-3S-specific IgG1^+^ GC (GL7^+^IgD^Low^) and NGC (GL7^-^IgD^+^) B cell isolations [chromium single cell V(D)J assay].

### Gene Expression Analysis of Single Cells

A total of 747 single cells were analyzed from Alum conditions (n = 375 cells) and SQE conditions (n = 372 cells) across W3 and W5. Single-cell gene expression analysis was performed using the BioMark 96.96 Dynamic Array IFCs and the Biomark HD System from Fluidigm. Two-step single-cell gene delta gene expression was performed using EvaGreen Supermix according to Fluidigm real-time PCR protocol. Briefly, reverse transcription using a 2-step VILO cDNA Synthesis Kit (Invitrogen, Carlsbad, CA, USA) was performed directly on single cells prior to specific target cDNA amplification.

Probes for 96 genes were selected from the catalog of delta gene expression assay (Fluidigm; **Table S1**). Processing of the IFCs and operation of the instruments were performed according to the manufacturer’s procedures. Thirty cycles of PCR were performed using the Biomark microfluidic chip (Fluidigm). Automated data analysis was performed with the Singular Analysis Toolset (v3.6) compatible with R software. We merged data from two chips (of two experiments) per condition (n = 184–188 cells). A multidimensional scaling analysis was performed without significant difference between chips. Results of negative control (no cells) or positive control samples (10 cells) and probe controls or non-amplified probes were removed before analysis using UMAP regression, heatmap after standard normalization, FlowSOM Elbow Metaclustering, and volcano plot EdgeR analysis (OMIQ Data Science Platform).

### V(D)J Repertoire and Gene Expression Profiling

W614A-3S-specific IgG1^+^ GC and NGC B cells at W11 were loaded according to the manufacturer’s instructions for the chromium single-cell V(D)J reagent kits (10x Genomics) to attain between 500 and 10,000 cells per condition. Library preparation for V(D)J sequencing was performed according to the manufacturer’s protocol [chromium single-cell V(D)J enrichment kit, mouse B cells] prior to sequencing on the Illumina MiSeq. Quality of raw reads was assessed using the FastQC 0.11.8 quality control tool, and clonotype quantification was performed with the Cell Ranger 3.1.0 V(D)J pipeline. Only 433 clonotypes defined as IgG protein sequences with exactly one heavy and one light chain were considered in the analysis. CDRH3 and CDRL3 sequence alignments were performed with Unipro UGENE software.

### Monoclonal Antibody Production

V(D)J sequences of W614A-3S-specific GC and NGC B cells were chosen from squalene conditions. Sixteen monoclonal antibodies were produced by the ProteoGenix company. An endotoxin-free DNA preparation was made for the pTXs1 expression constructions prior to XtenCHO™ transient expression. The recombinant Abs were then purified by affinity vs. protein G. After production, specificity tests by ELISA and a neutralization assay were performed.

### Statistics

Statistical analyses and graphical representation were performed using either GraphPad Prism 9, FlowJo X, OMIQ, Unipro UGENE software, or R 3.6.2. A two-way ANOVA test was used for kinetic peptide-specific IgG evaluation. Mann–Whitney tests were used to compare SQE and Alum conditions.

### Study Approval

All experimental protocols were approved by the French animal experimentation and ethics committee and validated by Service Protection et Santé Animales, Environnement, with the numbers 00954.02 and APAFIS#5863–2016062710255883 v3 for mouse experiments.

## Results

### Squalene Emulsion Adjuvant Is More Effective Than Alum at Eliciting Broadly Neutralizing Antibodies in Response to W614A-3S Peptide-Conjugated Vaccine

**Figure 1:**
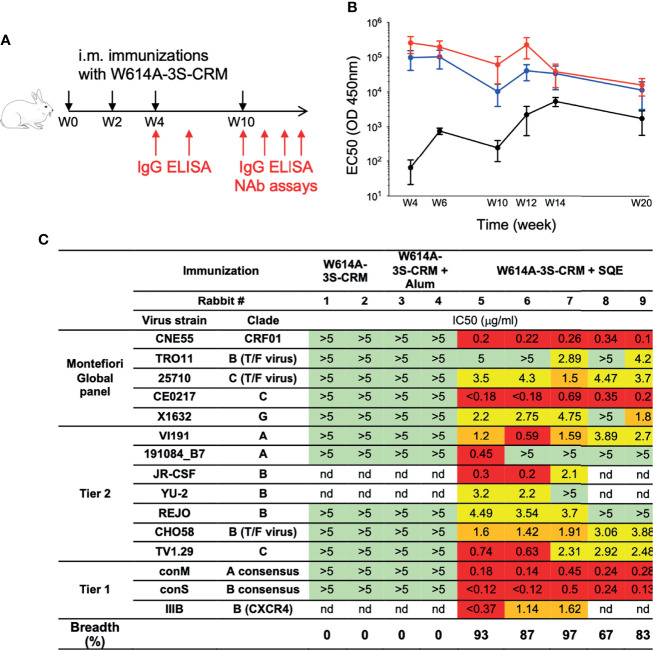
Broad neutralizing Ab induction after squalene emulsion-adjuvanted W614A-3S peptide vaccination. **(A)** Experimental schedule of rabbit immunizations and biologic samples. Animals were vaccinated at Week 0 (W0), W2, W4, and W10 (black arrows), and blood samples were collected at W4, W6, W10, W12, W14, and W20 (red arrows). **(B)** Rabbits were immunized with W614A-3S-CRM197 carrier non-adjuvanted (n = 4), adjuvanted with aluminum hydroxide (Alum) (n = 5), or squalene emulsion (SQE) (n = 5). Kinetics of serum W614A-3S-specific IgG of rabbits, immunized with W614A-3S-CRM197 non-adjuvanted (black), or adjuvanted with Alum (blue) or SQE (red). Graph represents EC50 mean ± SEM of OD 450 nm; the y-axis is in log 10 scale. Statistical analyses were performed with two-way ANOVA between two adjuvanted conditions. **(C)** Serum W614A-3S-specific IgG per condition (n = 2 for non-adjuvanted condition, n = 2 for Alum condition, and n = 5 for SQE condition) were purified at W20, and neutralizing IC50 were evaluated against different viruses including the Montefiori Global panel (16), Tier2, and Tier1. Color codes of neutralization assay (IgG μg/ml): green, >5; yellow, 4.9-2; orange, 1.9-1; and red, <0.9; nd: not done. Breadths represent percentage of total tested HIV strains neutralized by anti-W614A-3S IgG. T/F: transmitted/founder virus.

To evaluate the efficacy of W614A-3S-CRM197-conjugated vaccine, formulated in SQE and Alum, in inducing bNAbs, rabbits were immunized intramuscularly at week 0 (W0), W2, W4, and W10 in two independent experiments (n = 5 per condition) (**Figure 1A**). High levels of total W614A-3S-specific IgG Abs were detected in all rabbits regardless of the adjuvanted formulation (**Figure 1B**). We then assessed the neutralization activities against a panel of Tier 1 and Tier 2 viruses by TZM-bl neutralization assay using whole serum at W20 (two independent experiments, n = 2–5). Only W614A-3S-CRM197 immunization using the SQE formulation induced NAbs; however, this varied among rabbits (**Supplementary Figure 1**). The breadth of antibody neutralization varied between 25% and 69% of virus strains tested in rabbits. Therefore, purified W614A-3S-specific rabbit IgG was tested in the TZM-bl neutralization assay (**Figure 1C**). Out of a panel of 15 HIV virus strains tested, the SQE formulation induced W614A-3S-specific rabbit IgG that neutralized 67% to 93% of virus strains (**Figure 1C**). The majority of rabbit purified Abs neutralized five viruses of the Montefiori global panel used for standardized assessment of NAb efficacy (16).

**Figure 2:**
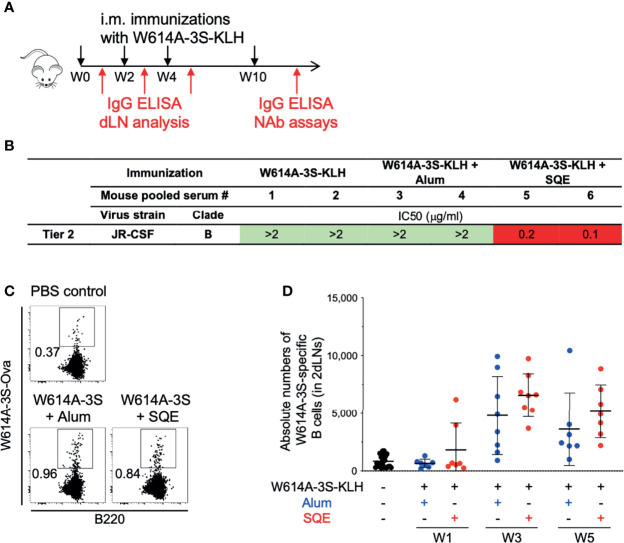
Validation of neutralizing Abs in mouse models and detection of W614A-3S-specific B cells. **(A)** Experimental schedule of mouse immunizations and biologic samples. Animals were vaccinated with W614A-3S-KLH carrier non-adjuvanted, adjuvanted with Alum or SQE at Week 0 (W0), W2, W4, and W10 (black arrows), and blood samples were collected or draining lymph node (dLN) analysis was made after each injection (red arrows). **(B)** Neutralizing IC50 of serum-purified W614A-3S-specific IgG was evaluated at W11 against Tier 2 virus JR-CSF strain. Purified peptide-specific IgG was obtained by pooling sera of 4–5 mice for each condition. Color codes of neutralization assay (IgG μg/ml): green, >2; orange, 1.9-1; and red, <0.9. **(C)** Representative dot plots of W614A-3S-specific B cells in dLNs after three immunizations with PBS (PBS control) or W614A-3S-KLH adjuvanted with Alum or SQE. Numbers represent peptide^+^ cell percentages of B cells. **(D)** A dot plot with mean ± SD (n = 7–23) represents the absolute numbers of W614A-3S-specific B cells in two dLNs, 1 week after first (W1), second (W3), and third (W5) immunizations with PBS (black), W614A-3S-KLH adjuvanted with Alum (blue) or SQE (red). Statistical analyses were carried out using the Mann–Whitney *U* test.

In order to further elucidate the immune mechanism of induction of NAbs versus non-NAbs using these two formulations, we validated our vaccine strategy using W614A-3S peptide, coupled with either the KLH or the CRM197 carrier with SQE or Alum formulation in the mouse model. The immunization protocol is shown in **Figure 2A**. W614A-3S-specific IgG was detected (by ELISA) in all mice immunized with W614A-3S peptide coupled with either CRM197 or KLH and formulated in SQE (Log10 EC50 mean ± SD: 4.31 ± 0.58 for CRM197 condition; 3.52 ± 0.19 for KLH condition) or Alum (Log10 EC50 mean ± SD: 4.31 ± 0.65 for CRM197 condition; 2.67 ± 0.44 for KLH condition) (data not shown). We then purified W614A-3S-specific IgG of mouse pooled serum in each condition. We confirmed detection of the neutralization of two Tier 2 viruses (JR-CSF and YU-2) by W614A-3S-specific Abs following W614A-3S-CRM197 (**Supplementary Figure 2**) or W614A-3S-KLH immunization (**Figure 2B**). Again, only the vaccine formulated with SQE emulsion and not with Alum induced NAbs responses.

We hypothesized a differential B cell maturation following vaccination with different adjuvant formulations. In order to study antigen-specific B cells, we measured W614A-3S-specific B cells in draining lymph nodes (dLNs), using W614A-3S-biotinylated ovalbumin (Ova). Flow cytometry analysis of W614A-3S-biotinylated Ova staining of B cells in either PBS or W614A-3S formulated in SQE or Alum is shown in **Figure 2C**. Despite background staining in control PBS-injected mice, we were able to identify the W614A-3S-specific B cells in mice over time with both the SQE and Alum formulations (**Figures 2C, D**). No significant difference in the absolute numbers (nor frequency; data not shown) of W614A-3S-specific B cells between SQE and Alum conditions was observed at both time points (**Figure 2D**).

In conclusion, we found that SQE formulation, W614A-3S conjugated with a carrier protein, induced bNAbs while the Alum formulation did not with a similar amount of W614A-3S-specific B cells. Here, we aim to elucidate the mechanism of B cell immune responses.

### Differential Gene Expression in W614A-3S-Specific B-Cell Populations Following Peptide-KLH Vaccination Using SQE and Alum Formulations

We used a single-cell quantitative reverse transcription-PCR (qRT-PCR) approach to compare between the two formulations the quality of W614A-3S-specific B cell populations isolated from dLNs, 1 week after the 2nd and 3rd immunizations (W3 and W5, respectively; **Figure 2C**).

**Figure 3:**
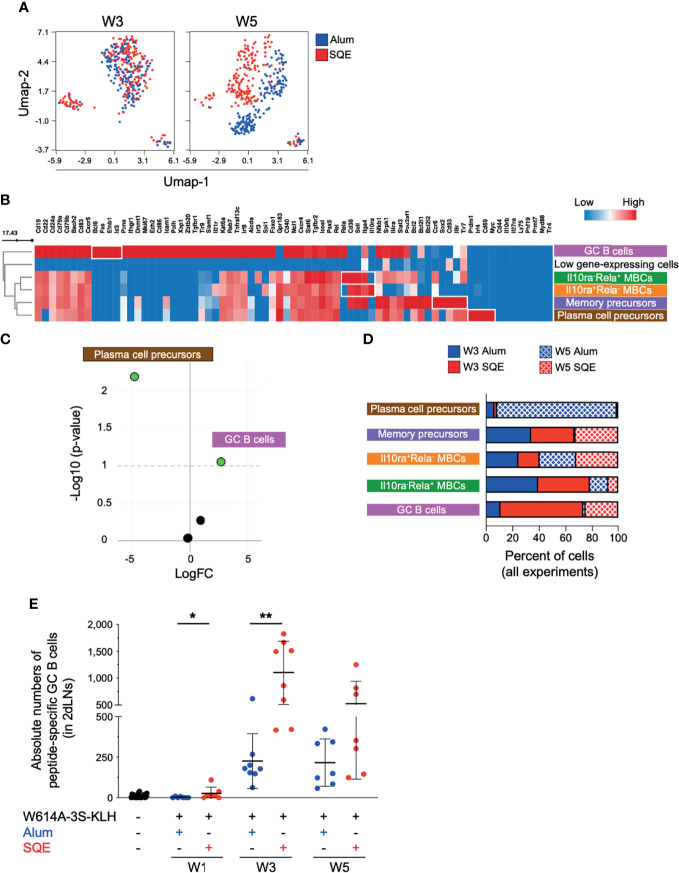
Differential W614A-3S-specific B cell populations after adjuvanted vaccination. Draining lymph nodes (dLNs; n = pool of 5 mice/condition - two independent experiments) were harvested at W3 and W5 after immunization and used to isolate peptide-specific B cells and to perform single-cell gene expression analysis. **(A)** Uniform manifold approximation and projection (UMAP) plots were generated by concatenation of 747 single cells from Alum (blue) and SQE (red) condition across W3 and W5 for expression of 73 genes. **(B)** Heatmap showing the medians of gene expression (all time points) for six different clusters, distinguished using the FlowSOM algorithm. One represented cluster with few cells (38 of 747 cells) and low gene expression was removed. Gradient color: lower (blue) to high (red) expression. B cell subpopulation names are indicated. **(C)** Volcano plot [log fold change (FC) versus -Log10 (p-value)] depicting five different clusters indicating empirical analysis of digital gene expression between adjuvanted conditions, with significant difference (p-value threshold <0.1; green points). **(D)** Graph represents cell percentages for each condition (Alum at W3, solid blue; SQE at W3 solid red; Alum at W5, wire blue; and SQE W5, wire red), based on 100% of evaluated cells [n = 97 plasma cell precursors; n = 224 memory-B cell precursors; n = 268 Il10ra^+^Rela^-^ memory B cells (MBCs); n = 64 Il10ra^-^Rela^+^ MBCs; and n = 56 germinal center (GC) B cells]. **(E)** A dot plot with mean ± SD (n = 7–13) represents the absolute numbers of W614A-3S-specific GC B-cells in two dLNs, at W1, W3, and W5. Statistical analyses were carried out using the Mann–Whitney *U* test and statistical significance is indicated between adjuvanted conditions: *p < 0.05; **p < 0.01.

We purified W614A-3S-specific B cells by cell sorting two groups of five mice per condition. Following quality control and data cleaning steps (M&M and **Supplemental Table 1**), we analyzed 73 detectable genes, in 747 antigen-specific B-single cells (184–188 cells per condition). We used dimensional UMAP (Uniform Manifold Approximation and Projection) regression to compare B lymphocyte distribution in SQE and Alum immunization conditions (**Figure 3A**), at W3 (2nd injection) and W5 (3rd injection). Whereas no difference in B cell distribution was observed at W3 (2nd injection), UMAP allowed segregation of B cells in the SQE formulation compared to the Alum formulation at W5 (3rd injection), suggesting that qualitative differences in W614A-3S-specific B cells would occur between the 2nd and 3rd injections.

A FlowSOM elbow metaclustering of all 747 cells allowed us to identify six populations of antigen-specific B cells that are represented by different levels of gene expression (**Figure 3B**). One cluster representing a few cells (38 of 747 cells) with very low gene expression was removed from the analyses. We identified GC B cells (high expression of Bcl6, Fas, Efnb1, and Id3 genes) and plasma cell precursors (high expression of Prdm1, Irf4, Cd69, and Myc genes). We also identified three different memory B-cell (MBC) populations (high expression of Cd38, Sell, and Itga4 genes) that are differentiated by 1) high expression of Ccr6, Il9r, Tlr7, Sox2, and Cd93 genes; 2) low expression of Rela and high expression of Il10ra; and conversely, 3) high expression of Rela and low expression of Il10ra genes. According to the literature, high-expressing CCR6 B cells might be memory precursors in the GC light zone with low antigen affinity (17). GC B cells and plasma cell precursor populations were individualized and compared to three MBC populations. The abundance of plasma cell precursors in Alum conditions and the abundance of GC B cells in SQE conditions were significant, as shown in a Volcano plot (p-value threshold <0.1; **Figure 3C**). The percent of total number of cells (n = 747) is represented in **Figure 3D**. We found that plasma cell precursors were present principally after the 3rd immunization (W5) in Alum conditions, whereas GC B cell numbers were higher in SQE conditions (**Figure 3D**). However, the proportions of the three MBC populations were relatively similar between Alum and SQE formulations (**Figure 3D**).

This increase in GC B cell number after SQE immunization over time was further validated by flow cytometry (**Figure 3E**). We observed significantly higher numbers of W614A-3S-specific GC B cells after the first and second injections (p = 0.0495 and 0.0011, respectively), as detected in SQE conditions compared to Alum conditions. When comparing single-cell analysis to flow cytometry, we found that both formulations induced GC B cells; however, their frequencies were significantly higher in SQE conditions compared to Alum, with similar gene expression profiles at W3. Thus, at W5, the gene expression of W614A-3S-specific B cells was distinct in SQE conditions compared to Alum with differential quantities of GC B cells in addition to qualitative gene expression differences following immunization using the two formulations.

### Significant Diversity of B-Cell Repertoire of Germinal Center and Non-Germinal Center B Cells Following W614A-3S-KLH Vaccination Using SQE and Alum Formulations

**Figure 4:**
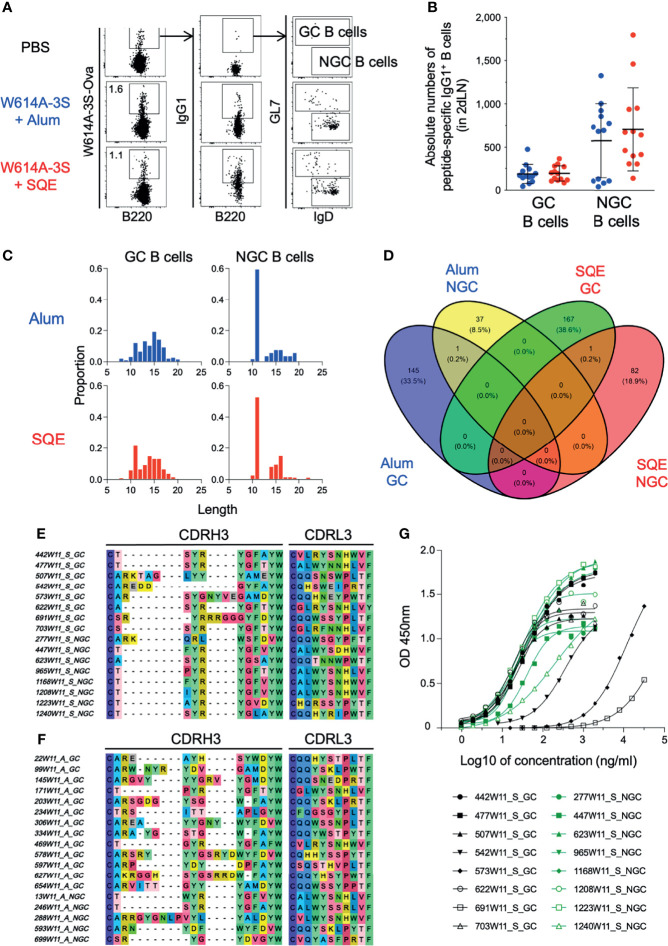
Repertoire diversity of germinal center and non-germinal center B cells after adjuvanted vaccination. **(A)** Representative dot plots of gating strategy for W614A-3S-specific IgG1^+^ germinal center (GC) and non-germinal center (NGC) B cells of dLNs sorting after four W614A-3S-KLH adjuvanted immunizations. Numbers represent peptide^+^ cell percentages of B cells. **(B)** A dot plot with mean ± SD (n = 7–13) represents the absolute numbers of W614A-3S-specific IgG1^+^ GC and NGC B cells in two dLNs at W11. Statistical analyses were carried out using the Mann–Whitney *U* test. For W614A-specific IgG1^+^ B-cell repertoire analysis, 2 dLNs of 25 mice per condition were pooled at W11. **(C)** Distribution of CDRH3 amino acid lengths for 433 clonotypes of GC B cells (left graphs) and NGC B cells (right graphs) for Alum condition (blue; upper graphs) and SQE condition (red; lower graphs). **(D)** Venn diagram of 433 CDRH3 (heavy chain) + CDRL3 (light chain) protein sequences (number and percentage) per condition (GC B cells of Alum condition, blue; NGC B cells of Alum condition, yellow; GC B cells of SQE condition, green; NGC B cells of SQE condition, red). Representative sequences of CDRH3 and CDRL3 of higher frequent clonotypes for SQE conditions **(E)** and Alum conditions **(F)**. The V(D)J sequences of these SQE clonotypes were used for production of 16 clonal IgG. **(G)** Anti-W614A-3S specificity of 16 clonal IgG1 titers (from SQE conditions) were measured by ELISA. Graph represents OD_450_ nm versus log10 of IgG1 concentration (ng/ml). Eight clonal IgG1 issued from GC B cells (black) or from NGC B cells (green) were produced and evaluated.

To evaluate the clonal diversity of W614A-3S-specific B cells using SQE and Alum formulations, we purified W614A-3S-specific B lymphocytes of dLNs at W11, 1 week after the last immunization. We purified W614A-3S-specific IgG1^+^ GC (GL7^+^IgD^-^) and non-GC (NGC; GL7^-^IgD^+^) B cells for single-cell V(D)J sequencing (the gating strategy is shown in **Figure 4A**). We did not find any significant difference in the absolute numbers of W614A-3S-specific IgG1^+^ GC and NGC B cells between SQE and Alum conditions (**Figure 4B**).

Amino acid analysis of the variable part of heavy (H) and light (L) IgG1 chain sequences, the third complementarity-determining region (CDR3), was carried out for both conditions and in cell types with a total of 433 distinct sequences, regardless of their frequencies in samples. Clonotypes with exactly one heavy and one light chain were considered in the analysis. The proportions of cells with different lengths of CDRH3 were relatively similar in Alum and SQE conditions for GC or NGC B lymphocytes (**Figure 4C**). Venn diagram analysis showed the number of CDR3 (H+L) sequences in each condition (SQE and Alum) and cell type (GC and NGC B cells) (**Figure 4D**). We found one common clone with sequence similarities between GC and NGC cell populations in each SQE (H chain: CSSYRYGLGYW; L chain: CGLWYGNHWVF) and Alum (H chain: CTSYRYGFAYW; L chain: CALWYSSRLVF) conditions. We represented the most frequent CDRH3 and CDRL3 sequences in the SQE condition compared to the Alum function (**Figures 4E, F**, respectively). Interestingly, sequences generated in SQE were distinct from the one generated in the Alum condition, demonstrating the major differences in amino acid alignment between formulations.

In order to verify if the Ab sequences were specific to W614A-3S, we selected 16 majorly represented out of 433 clonotypes (GC and NGC B cells). We cloned and produced recombinant monoclonal IgG1 with completed V(D)J sequences among the most frequent ones in SQE conditions. We confirmed that selected monoclonal Abs were specific to the W614A-3S peptide (**Figure 4G**). However, some of them (e.g., clone 691W11_S_GC, 573W11_S_GC) showed lower affinity. None of these selected, cloned, and produced peptide-specific monoclonal Abs were able to neutralize HIV (data not shown). Despite this disappointing score, the single-cell V(D)J sequencing of W614A-3S-specific BCR validated the dichotomy in BCR sequences between SQE and Alum formulation.

Altogether, both approaches using single-cell analysis of gene expression and BCR sequencing revealed that even though W614A-3S-specific B cells (IgG1^+^) were detected following injection of both formulations, gene expression, B cell differentiation, and BCR selection were distinct.

## Discussion

This work is focused on preclinical immunogenicity of a modified gp41 peptide “NH2-pwnaSASNKSlddiw-COOH,” with a single amino acid change at position W614 to alanine (namely, W614A-3S). We demonstrated that the formulation of W614A-3S-carrier protein with either Alum or SQE impacts the quantity and quality of B lymphocytes. These differences result in 1) the induction of non-NAbs for the Alum formulation versus the production of NAbs for the SQE formulation, which were capable of neutralizing a wide range of HIV-1 strains including Tier 1 and Tier 2 viruses (73%–93% breadth); 2) major differences in the proportion of differentiated B cells expressing GC B-cell-associated genes; and 3) a distinct amino acid sequence of the CDR3 region. One limitation is the paucity of these bNAbs in the serum of animals. Indeed, rare neutralizing activities were detected using the whole sera of rabbits or mice; a purification step of peptide-specific IgG1 was necessary to detect virus neutralization. The dichotomy between Alum and SQE formulation is intriguing and strengthens our results.

BNAbs generally arise late in the course of HIV infection. The most mature bNAbs revealed one or more unusual features, such as high levels of somatic hypermutation, unusually long complementary-determining regions like CDRH3, and polyreactivity for non-HIV-1 antigens (18–20). While it is difficult to establish the generality of these observations, and if these features are required for the development of bNAbs, these features reveal a long and intensive process of B cell selection. It is necessary to elicit a similar Ab response by vaccination strategies. We have shown that anti-W614A-3S NAbs were rarely observed in HIV-1 progressors but were significantly increased in untreated, long-term non-progressor patients of the ALT ANRS cohort who were infected for more than 7 years but did not develop the disease (11). These anti-W614A-3S Abs are found in strong association with both viral load and viral DNA and are able to neutralize most of the HIV-1 clade B tested. HIV vaccine studies are currently based on the Env protein in a trimeric form. However, other approaches have provided evidence for a peptide epitope-based vaccine (21–23). The recent discovery of Abs with neutralizing breadth against the fusion peptide (FP) may lead to promising vaccine candidates (6). In mice, the FP elicited monoclonal Abs capable of neutralizing 31% of a cross-clade panel of 208 HIV-1 strains. The crystal structure and cryoelectron microscopy structures of these antibodies revealed FP diversity as a molecular explanation of cross-clade neutralization in macaque and guinea pigs (6).

We have chosen two main adjuvants that can be carried over into human clinical studies, Alum and SQE, as both are based on licensed human product formulations. Our SQE formulation is based on AddaVax™, which is a squalene-based, oil-in-water nano-emulsion based on the formulation of MF59^®^ that has been licensed in Europe for adjuvanted flu vaccines (24). MF59^®^ increases GC B cell differentiation and also induces persistent high-affinity functional Ab titers (25). In the context of pandemic influenza vaccine, MF59^®^ adjuvant stimulates induction of broadly cross-reactive Abs (26). SQE formulation induced bNAbs with 65%–90% breadth of neutralization of Tier 1 and Tier 2 HIV strains, whereas the Alum formulation induced Abs without detectable neutralizing activities. This class of adjuvant is believed to act through a depot effect, enhancing antigen persistence at the injection site, recruiting and activating antigen-presenting cells, and directly stimulating cytokine and chemokine production by macrophages and granulocytes (24).

We further investigated the impact of Alum and SQE formulations of the W614A-3S vaccine on B cell differentiation. In non-human primates, higher frequencies of total and Env-specific GC-Tfh cells accompanied by larger and more diverse Env-specific B cell lineages were found after slow delivery of antigen (27). It has also been proposed that squalene oil-in-water emulsion facilitates a rapid Ab response in contrast to aluminum hydroxide due to different kinetics of delivery of antigen to the lymphoid organs (28). In the influenza vaccine, squalene-based emulsion adjuvants increase Ab affinity against the hemagglutinin-based vaccine and breadth of B cell responses, leading to protection across virus clades (29).

We used a single-cell qRT-PCR approach to compare the gene expression of W614A-3S-specific single-B cells between the two formulations. We identified GC B cells (high expression of Bcl6 (30), Fas (31), Efnb1 (32), and Id3 (33)), plasma cell precursors (high expression of Prdm1, Irf4, Cd69, and Myc), and three different MBC populations according to the literature definition (34–36). Our data showed that the proportion of GC B cells as defined by single-cell gene expression was significantly higher when the W614A-3S vaccine was formulated in SQE, whereas plasma cell precursors were significantly higher following Alum formulation. Thus, qualitative and quantitative differences in the proportion of differentiated B cells were revealed by single-cell analyses.

The single-cell V(D)J sequencing of W614A-3S-specific BCR validates the dichotomy between SQE and Alum formulations. Significant diversity of the B cell repertoire of GC and NGC-B cells was observed following W614A-3S vaccination using SQE compared to Alum formulations. We did not find any neutralizing activities among cloned Abs according to the defined sequence. There are some limitations in our study, notably in regard to BCR sequencing for detection of NAbs. Indeed, we used W614A-3S peptide conjugated with biotinylated ovalbumin in order to purify antigen-specific B cells that might impact the selection of certain BCR during this step. Of note, vaccination of animals with a W614A-3S peptide in IFA induces neutralizing anti-HIV-1 Abs, among which we found a unique clone, F8, by hybridoma generation (37), suggesting the rarity of B cell clones or technical challenges in purification of B cells.

We found a dichotomy in the induction of bNAbs, B-cell gene expression, and BCR sequence after immunization using W614A-3S formulated in SQE compared to Alum. Our study challenges HIV vaccine development and discovery of formulation that could program B-cell differentiation favoring bNAbs. These data have been used in Go/No-go criteria for a clinical study. Indeed, an experimental medicine study using W614A-3S-CRM197 vaccine candidate (VAC02, Minka Therapeutics, France) is ongoing with the hope to speed up HIV vaccine research (ClinicalTrials.gov NCT04753892).

## Data Availability Statement

The original contributions presented in the study are publicly available. This data can be found here: www.ncbi.nlm.nih.gov/geo/query/acc.cgi?acc=GSE198883; https://www.ncbi.nlm.nih.gov/geo/query/acc.cgi?acc=GSE198952.

## Ethics Statement

The animal study was reviewed and approved by Service Protection et Santé Animales, Environnement.

## Author Contributions

BC and VV designed the study. OB, CC, MT, MB, LB, and VV carried out the experimental work and contributed to the data acquisition, analysis, and interpretation. SB, KA, and JN compiled and analyzed the single-cell repertoire data. ES and DK were responsible for adjuvant production and quality control. MB and GS were responsible for the neutralization assays and analysis. BC, PD, GS, DK, and VV provided financial support. BC and OB wrote the manuscript. All authors contributed to the article and approved the submitted version.

## Funding

This project has received funding from the European Union’s Horizon 2020 research and innovation program under grant agreement no. 681137. BC’s laboratory has received funding from the Fondation pour la Recherche Médicale “Equipe FRM 2020” award. GC’s laboratory thanks the Fondation Dormeur, Vaduz, for funding laboratory instruments relevant to this project. High-throughput sequencing has been performed by the ICGex NGS platform of the Institut Curie supported by the grants ANR-10-EQPX-03 (Equipex) and ANR-10-INBS-09-08 (France Génomique Consortium) from the Agence Nationale de la Recherche (“Investissements d’Avenir” program), by the Canceropole Ile-de-France, and by the SiRIC-Curie program – SiRIC Grant “INCa-DGOS-4654”.

## Conflict of Interest

VV is a member of the scientific advisory board of Minka Therapeutics. ES and DK were employed by Polymun Scientific Immunbiologische Forschung GmbH. SB, KA, and JN were employed by Altrabio.

The remaining authors declare that the research was conducted in the absence of any commercial or financial relationships that could be construed as a potential conflict of interest.

## Publisher’s Note

All claims expressed in this article are solely those of the authors and do not necessarily represent those of their affiliated organizations, or those of the publisher, the editors and the reviewers. Any product that may be evaluated in this article, or claim that may be made by its manufacturer, is not guaranteed or endorsed by the publisher.

## Abbreviations

Alum, aluminum hydroxide; bNAb, broadly neutralizing antibody; GC, germinal center; NGC, non-germinal center, SQE, squalene emulsion.
