# Supplementary material for: Dichotomy in Neutralizing Antibody Induction to Peptide-Conjugated Vaccine in Squalene Emulsion Contrast With Aluminum Hydroxide Formulation
Source: Front Immunol. 2022 Apr 7;13:848571. doi: 10.3389/fimmu.2022.848571 (PMC9021396; doi:10.3389/fimmu.2022.848571)
Supplement: Supplementary file 1 [file DataSheet_1.pdf]

Dichotomy in neutralizing antibodies induction to peptide-conjugated vaccine in squalene emulsion contrast with aluminum hydroxide formulation

Olivia Bonduelle, Chloé Chaudesaigues, Monica Tolazzi, Ehsan Suleiman, Simon de Bernard, Karine Alves, Julien Nourikyan, Mylene Bohec, Laura G. Baudrin, Dietmar Katinger, Patrice Debré, Gabriella Scarlatti, Vincent Vieillard, Behazine Combadière.

Supplementary Table

Supplemental Table 1: List of 73 genes and probe sequences used for the interrogation of peptide-specific single B cells. Related to Figure 3.

Gene Symbol	Forward Primer	Reverse Primer
**Aicda**	CCTGGGAAGGGCTACATGAAA	CGCAAGTCATCGACTTCGTAC
**Bach2**	GGTTGGACAGACGAAAGATGAC	AACTGTAGCAGTGGCCCAAA
**Bcl2**	ATGTGTGTGGAGAGCGTCAA	GATGCCGGTTCAGGTACTCA
**Bcl2l1**	AGCGTAGACAAGGAGATGCA	AGGCTCTAGGTGGTCATTCA
**Bcl2l2**	GTCCTAAGAGCTGCCATCCA	AGCCTACAAAGTCAGCCACTA
**Bcl6**	GGGGAAACCCAGTCAGAGTA	CTCAGAGAAACGGCAGTCAC
**Ccr6**	AAGGCACATATGCGGTCAAC	CCTGGACGATGGCAATGTAC
**Cd19**	CCATCGAGAGGCACGTGAA	ACCACTGGGACTATCCATCCA
**Cd22**	CTCTTGCTTGGCAGAGAACC	GCTCTGAATCACTGTGGTCAC
**Cd24a**	GCAGATCTCCACTTACCGAAC	CAGTGCCAGAAGCAGCAA
**Cd28**	CTGCTGTTCTTGGCTCTCAAC	GGGCGACTGCTTTACCAAAA
**Cd38**	AGACTACGCCCCACTTGTTA	CAGGTGTTTGGATTTGCTCCA
**Cd40**	CTATGGGGCTGCTTGTTGAC	TCGTGGAGGTACTGTTTGTCA
**Cd44**	TTCCTTCGATGGACCGGTTA	TACTCGCCCTTCTTGCTGTA
**Cd69**	GTGGTCCTCATCACGTCCTTA	ACAAGCCTGGGCAATTGTAC
**Cd79a**	AAGAACCACAGGGGCTTGTA	CATGTCCAGGAAGGGCCTA
**Cd79b**	TTCATCATTGTGCCCATCTTCC	AGGTGTGATCTTCCTCCATCC
**Cd83**	CCGCAGCTCTCCTATGCA	AGCTGTTTTGCTTGCTCTCC
**Cd86**	CATGGGCTTGGCAATCCTTA	CATTGAAATAAGCTTGCGTCTCC
**Cd93**	ACAGCTATTCCTGGGTTCCA	AGCTGTCTCTAAGGCCACATA
**Cxcr4**	GGTAACCACCACGGCTGTA	CAGGGTTCCTTGTTGGAGTCA
**Cxcr5**	GGACATGGGCTCCATCACATA	TCCCTCGACTGTAGAGCAGAA
**Dnmt1**	AGCCATTGGCCTGGAGATTA	GCAGCCTCCTCTTTTGCTTTA
**Efnb1**	CGTGTCCTGGAGCTCTCTTAA	TGATGTCCAGCTTGTCTCCAA
**Ezh2**	TGATGGAAAAGTGCATGGTGAC	GACCAAGAGCATTTACCAACTCC
**Fas**	TGTTTTCCCTTGCTGCAGAC	CCGCCTCCTCAGCTTTAAAC
**Foxo1**	GAAGAGCGTGCCCTACTTCA	GGACAGATTGTGGCGAATTGAA
**Gpr183**	CAAACACGGACTGCCACAAC	GACGTTGCCAGTGGGGTA
**Icam1**	AGGGCTGGCATTGTTCTCTA	TGTCGAGCTTTGGGATGGTA
**Icosl**	GGCTCTCCACCAGAACATCA	GGACGGGGACAAGGACTTTTA
**Id3**	TCCTGCAGCGTGTCATAGAC	CCTCTTGTCCTTGGAGATCACA
**Ifngr1**	CTGGGAATACCAGAACATGTCAC	TGCAGGAATCAGTCCAGGAA
**Il10ra**	AACTGGACCACCACTGAGAC	GCTTTCAGAGTCACGCTATCC
**Il10rb**	AACAGGAGAGTGGAGTGAACC	TGATGGCCACAATCCAGGAA
**Il21r**	AGTTTCTTCCAGCCCCTGTA	AGGCCGTGAAAGGGGTATTA
**Il27ra**	ACAAGGAATGCCAGGCTGAA	GGTTCCAGGTTCTGCATCTCA
**Il4ra**	AACATCTCCAGAGAGGACAACC	CTCAGCCTGGGTTCCTTGTA
**Il9r**	GCTACGAGCTGGCCTTCAA	GCCAGGTCACTCCAACGATA
**Irf3**	TCCTGACACCAATGGCAAAA	CCCAAGATCAGGCCATCAAA

**Irf4**	TCCCCATTGAGCCAAGCATA	CGAGGATGTCCCGGTAATACA
**Irf8**	GATATGCCGCCTATGACACA	CCCGTAGTAGAAGCTGATGAC
**Itga4**	TTCCAGAGCCACACCCAAAA	GATGATGGAGCCCTTCCAAGAA
**Kat6a**	CTGTCGTGACCAAGGCAAAA	GGTCACAACACTCCATGTGAAA
**Ly75**	ATGGAGCCGGAGTTTCAACA	GACTCTTCGCCAAGGTACATCA
**Mcl1**	AAACGGGACTGGCTTGTCA	AGGTCCTGTACGTGGAAGAAC
**Mki67**	GAGACATACCTGAGCCCATCA	GCTTTGCTGCATTCCGAGTA
**Myc**	AGTGCTGCATGAGGAGACA	TCTCCACAGACACCACATCAA
**Myd88**	GCCTTGTTAGACCGTGAGGATA	TCCTGGTTCTGCTGCTTACC
**Nfkb1**	ACCGTATGAGCCTGTGTTCA	GTAGCCTCGTGTCTTCTGTCA
**Pax5**	GCCACCCTCAGTATTCTTCCTAC	GGCTGCAGGGCTGTAATAGTA
**Pcna**	GACTTAGATGTGGAGCAACTTGGA	AAATTCACCCGACGGCATCT
**Phf19**	TCCATGAGGCTTGCACACA	ACACACGGAGCAGAAGAACA
**Polh**	TGACCCAGTTCACTGAATCCC	ACCTCGACACATGGCATACA
**Pou2af1**	AGAAGAAAGCGTGGCCATAC	GGCTGGTGGGGCAGTA
**Prdm1**	CGTTCGGTCAGCTCTCCAA	TGCAGGTCTGGCACTTGAAA
**Prmt7**	GTGTGGTACAGCCTTCAGAGAA	GAGCCTGACAGTCACACACA
**Rab7**	GGTGATGGTGGACGACAGAC	GCCACACCAAGAGACTGGAA
**Rel**	CTGCCTCCCATTGTTTCTAACC	CTTGTTCACACGGCAGATCC
**Rela**	CCAGCGAATCCAGACCAACA	AGAGGCGCACTGCATTCAA
**Sell**	CAGAGAGACTTGCAGAGAGACC	TCCTCGAGCCCCAGTAAGTA
**Spi1**	AACAGATGCACGTCCTCGATA	CATCCGGGGCATGTAGGAA
**Slamf1**	GCAATTCTGCAAGCAGCTGAA	AGGTCCCATTCTCGTTCTCC
**Sox2**	CGTAAGATGGCCCAGGAGAA	CTCGGACAAAAGTTTCCACTCC
**Srpk1**	ACAGACCCCTGACAGAGAAC	TCCTGGCCAATGGAATTTGAC
**Stat3**	TGGGCATCAATCCTGTGGTA	CCAATTGGCGGCTTAGTGAA
**Stat6**	TGACTTTCCACAACGCCTAC	CATCTGAACCGACCAGGAAC
**Tgfbr1**	AATTGCTCGACGCTGTTCTA	ACCGATGGATCAGAAGGTACA
**Tgfbr2**	TCTGTGAGAAGCCGCATGAA	GGCAAACCGTCTCCAGAGTAA
**Tlr4**	GTTCTTCTCCTGCCTGACAC	GCTGAGTTTCTGATCCATGCA
**Tlr7**	CCAAAGCACGCAGCTCAAA	CTGAGAAGGGAGCCAAGGAC
**Tlr9**	CGGGAGAATCCTCCATCTCC	CCTGTACCAGGAGGGACAA
**Tnfrsf13c**	TTCGACCCTCTGGTGAGAAAC	AGCTGTCCCAGGCTCCA
**Xbp1**	CAGCAAGTGGTGGATTTGGAA	CAAGGCCGTGAGTTTTCTCC
**Zbtb20**	ACAAACTCTCACGCTCACAC	CGAGCACGGAATTGCTGAA

Dichotomy in neutralizing antibodies induction to peptide-conjugated vaccine in squalene emulsion contrast with aluminum hydroxide formulation

Olivia Bonduelle, Chloé Chaudesaigues, Monica Tolazzi, Ehsan Suleiman, Simon de Bernard, Karine Alves, Julien Nourikyan, Mylene Bohec, Laura G. Baudrin, Dietmar Katinger, Patrice Debré, Gabriella Scarlatti, Vincent Vieillard, Behazine Combadière.

Supplementary Figures

	Immunization		W614A-3S-CRM		W614A-3S-CRM + Alum		W614A-3S-CRM + SQE				
	Rabbit #		1	2	3	4	5	6	7	8	9
	Virus strain	Clade	IC50 (1/dilution)								
Montefiori Global panel	CNE55	CRF01	<20	<20	<20	<20	305	230	117	<20	40
	TRO11	B (T/F virus)	<20	<20	<20	<20	21	26	<20	<20	<20
	25710	C (T/F virus)	<20	<20	<20	<20	32	20	<20	30	<20
	CE0217	C	<20	<20	<20	<20	152	156	151	334	408
	X1632	G	<20	<20	<20	<20	33	36	30	<20	25
Tier 2	VI191	A	<20	<20	<20	<20	69	152	86	<20	37
	191084_B7	A	<20	<20	<20	<20	105	89	36	<20	<24
	REJO	B	<20	<20	<20	<20	73	99	92	<20	<20
	CHO58	B (T/F virus)	<20	<20	<20	<20	62	124	51	<20	34
	TV1.29	C	<20	<20	<20	<20	54	106	81	<20	30
Tier 1	conM	A consensus	<20	<20	<20	<20	237	351	409	201	325
	conS	B consensus	<20	<20	<20	<20	104	237	145	362	373
	IIIB	B (CXCR4)	nd	nd	nd	nd	31	<20	<20	nd	nd
Breadth (%)			0	0	0	0	69	69	61	25	25

Supplementary Figure 1: Rabbits were immunized with W614A-3S-CRM197 carrier non adjuvanted (n=2), adjuvanted with Alum (n=2) or SQE (n=5) at W0, W2, W4 and W10. Whole serum W614A-3S specific Abs of 2 to 5 rabbits per condition were analyzed at W20, and neutralizing IC50 were evaluated against different virus strains including Montefiori Global panel, Tier2 and Tier1. Color codes of neutralization assay (1/dilution): green, ≤40; yellow, 41-100; orange, 101-200 and red, >201; nd: not done. Breadths represent percentage of total tested HIV strain neutralized by anti-W614A-3S IgG. T/F: Transmitted Founder virus.

	Immunization		W614A-3S-CRM + Alum	W614A-3S-CRM + SQE
	Mouse pooled serum #		1	2
	Virus strain	Clade	IC 50 (µg/ml)	
Tier 2	JR-CSF	B	>2	0.4
	YU-2	B	>2	1.9

Supplementary Figure 2: Mice were immunized with W614A-3S-CRM197 carrier adjuvanted with Alum or SQE at Week 0 (W0), W2, W4 and W18. Neutralizing IC50 of serum purified W614A-3S-specific IgG were evaluated at W20 against two Tier 2 virus strains (JR-CSF and YU-2). Purified peptide-specific IgG were obtained by pooling sera of 5 mice for each condition. Color codes of neutralization assay (IgG µg/ml): green, >2; orange, 1-1.9 and red, <0.9.

	Control mAb<br>Env region		10E8<br>MPER	2F5<br>MPER	PG9<br>V1V2 glycan	10-1074<br>V3 Glycan
	Virus strain	Clade	IC50 (µg/ml)			
Montefiori Global panel	CNE55	CRF01	>5	nd	0.26	nd
	TRO11	B (T/F virus)	>5	nd	>5	5.9
	25710	C (T/F virus)	3.86	>5	0.25	>5
	CE0217	C	>5	nd	<0.02	>1
	X1632	G	>5	nd	0.32	nd
Tier 2	VI191	A	1.96	>5	0.48	nd
	191084_B7	A	>5	nd	<0.02	nd
	REJO	B	>5	>5	0.06	>5
	CH058	B (T/F virus)	1.17	nd	>5	nd
	TV1.29	C	1.68	6.9	<0.02	>5
Tier 1	conM	A consensus	0.27	nd	0.04	0.46
	conS	B consensus	1.19	0.35	0.08	nd
	IIIB	B (CXCR4)	nd	>5	>1	0.1

Supplementary Figure 3: Control monoclonal Abs binding to different Env regions. Neutralizing IC50 were evaluated against different virus strains including Montefiori Global panel, Tier2 and Tier1. Color codes of neutralization assay (monoclonal Abs µg/ml): green, >5; yellow, 5-2; orange, 1.9-1 and red, <0.9; nd: not done. MPER: Membrane-Proximal External Region; T/F: Transmitted Founder virus.
